# Spectrum of Germline *RET* variants identified by targeted sequencing and associated Multiple Endocrine Neoplasia type 2 susceptibility in China

**DOI:** 10.1186/s12885-021-08116-9

**Published:** 2021-04-07

**Authors:** Xiao-Ping Qi, Jian-Qiang Zhao, Xu-Dong Fang, Bi-Jun Lian, Feng Li, Hui-Hong Wang, Zhi-Lie Cao, Wei-Hui Zheng, Juan Cao, Yu Chen

**Affiliations:** 1 Department of Oncologic and Urologic Surgery, The 903rd PLA Hospital, Wenzhou Medical University, 40 Jichang Road, Hangzhou, 310004 Zhejiang Province China; 2 Department of Head and Neck Surgery, Institute of Cancer Research and Basic Medical of Chinese Academy of Sciences, Cancer Hospital of University of Chinese Academy of Sciences, Zhejiang Cancer Hospital, 1 East Banshan Road, Hangzhou, 310022 Zhejiang Province China

**Keywords:** *RET* proto-oncogene, Multiple endocrine neoplasia type 2, Medullary thyroid cancer, Pheochromocytoma, Hyperparathyroidism, Genetic variants

## Abstract

**Background:**

Germline *RET* mutations and variants are involved in development of multiple endocrine neoplasia type 2 (MEN2). The present study investigated a spectrum of *RET* variants, analyzed genotype-phenotype relationships, and evaluated their effect on the MEN2 phenotype in Han Chinese patients.

**Methods:**

Targeted sequencing detected germline *RET* variants in 697 individuals, including 245 MEN2, 120 sporadic medullary thyroid cancer (MTC), and 15 pheochromocytoma (PHEO) patients and their 493 relatives. In silico analyses and classifications following ACMG-2015 were performed. Demographic, clinical variant types, and endocrine neoplasia molecular diagnosis records were also analyzed.

**Results:**

Nineteen different *RET* mutations (18 point and 1 del/ins mutations) in 214 patients with MEN2A (97.7%) or MEN2B (2.3%) were found, of which exon 11/10 mutations accounted for 79% (169/214). Nineteen compound mutations were found in 31 patients with MEN2A. Twenty-three variants (18 single and 5 double base substitution/compound variants) non-classification were also found. Of these, 17 (3 of pathogenic, 10 of uncertain significance, 2 of likely benign and 2 as benign) were found in 31 patients with MTC/PHEO. The remaining 6 variants (4 of uncertain significance and 2 of likely benign) found in 8 carriers had no evidence of MEN2. The entire cohort showed MEN2A-related PHEO, all occurring in exons 11/10, particularly at C634. Kaplan-Meier curves showed age-dependent penetration rates of MTC and PHEO, and occurrence rates of PHEO in patients with exon 11 mutations were all higher than those within exon 10; these bilateral PHEO were always associated with exon 11 mutations (all *P* < 0.05). While patient offspring had PHEO, parents with MEN2A had none, the frequency was approximately 10%. Interestingly, at least 6.8% of families were adoptive. Also, 3 non-hotspot *RET* variants (R114H, T278N, and D489N) appeared with high frequency. Conversely, polymorphism S836S was absent.

**Conclusions:**

These data are largely consistent with current evidence-based recommendations in the clinical practice guidelines. Diversity of *RET* variants or carriers may involve a different natural disease course. Further large-scale targeted sequencing studies will serve as an accurate and cost-effective approach to investigating MEN2 genotype-phenotype correlations for discovery of rare or unknown variants of *RET*.

## Background

Multiple endocrine neoplasia type 2 (MEN2) is a neuroendocrine cancer syndrome characterized by the propensity to develop medullary thyroid carcinoma (MTC) with or without pheochromocytoma (PHEO), hyperparathyroidism (HPTH), and extra-endocrine features, such as Hirschsprung’s disease (HD) or cutaneous lichen amyloidosis (CLA) [1]. MEN2 syndrome originating from the neural crest includes two clinically distinct forms subtyped as MEN2A (OMIM 171400; ~ 95% of MEN2) and MEN2B (OMIM 162300; ~ 5%) [1]. Nearly all MEN2 cases are caused by germline gain-of-function mutations of the *RET* proto-oncogene (OMIM 164761), except for two families who reportedly had germline mutations in *ESR2* or *MET* that predisposed them to MTC [1–3]. The identification of *RET* mutations as the cause of MEN2 has significantly changed MEN2 disease management, including disease prevention, risk prediction, early diagnosis, and personalized treatment of MEN2-specific tumors. Together, these approaches represent a paradigm of precision medicine [1, 4–9].

The *RET* proto-oncogene contains 20 exons and encodes a tyrosine kinase transmembrane receptor (https://www.ncbi.nlm.nih.gov/gene/5979). Sequencing of *RET* has resulted in the identification of 199 variants, of which approximately 45% are pathogenic mutants involved in MEN2 [10], and thus far, mutation hotspots are known to mainly occur in exons 8, 10, 11, and 13–16 [1, 9–17]. Based on the specific *RET* mutation (genotype) associated with phenotypes, the Revised American Thyroid Association (ATA) Guidelines (hereafter referred to as ATA-2015), among others, have defined the management of MTC risk category (moderate risk, ATA-MOD; high risk, ATA-H; and highest risk, ATA-HST) of each specific *RET* mutation and recommended the optimal age of prophylactic total thyroidectomy, timing by which patients carrying germline *RET* mutations should be screened for PHEO and HPTH, penetrance estimations, and surgical windows of opportunity [1, 18, 19]. Over the past 25 years, the prognosis of MEN2 patients has greatly improved, with disease-specific survival rate 20 years after prophylactic thyroidectomy of 89.9, 93.2, and 54.6% for MEN2 with ATA-MOD, ATA-H, and ATA-HST *RET* mutations, respectively. In addition, appropriate surgical procedures have also helped reduce MEN2-related postoperative complications [20–24]. However, previous studies have shown that the same *RET* mutation can result in different clinical phenotypes or disease course even among family members [1, 7, 11–17, 25, 26]. Besides primary driver *RET* mutations, some studies have indicated the potential effect of modifying factors (genetic or environmental) on risk of age-related PHEO penetrance and MTC aggressiveness in MEN2, such as *RET* single nucleotide polymorphisms [SNPs] [16, 27–31] or specific haplotypes [32]. Previous studies, however, were either mostly limited to *RET* SNP hotspots, reported controversial conclusions, or had not examined susceptibility [33–36]. These issues suggest the need for analysis of potential modifying factors, including the entire *RET* coding region, and an individualized strategy for clinical management of MEN2 should be considered [1, 19, 29, 34, 37, 38].

In the present study, a spectrum of germline *RET* variants were investigated in patients in our hospital using targeted genes and next-generation sequencing (targeted sequencing). Specific analysis of genotype-phenotype relationships, and the possible effects of rare *RET* variants on the MEN2 phenotype was assessed.

## Methods

### Participants

**Fig. 1:**
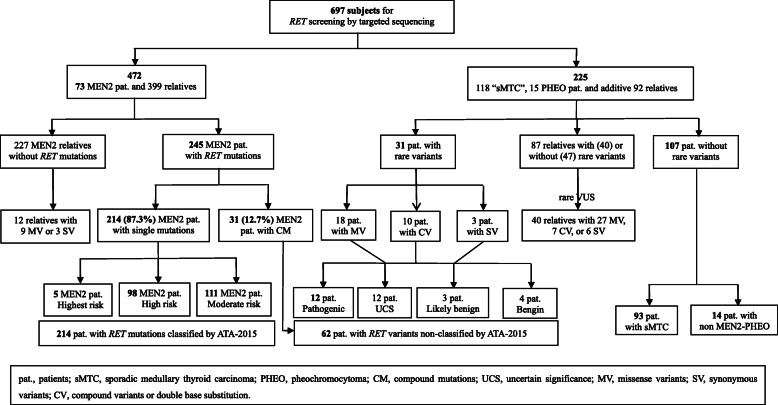
Pathogenic mutations or VUS in *RET* for 697 subjects including MTC/PHEO cases and screened relatives by targeted sequencing. MTC risk category following the ATA-2015 and proposition of classifying variants not found in the ATA-2015 using the consensus recommendation of the ACMG-2015

From 2011 to 2020, 206 index cases of histopathologically-diagnosed MTC and/or PHEO were subjected to genetic screening at the 903rd PLA Hospital and Zhejiang Cancer Hospital (Hangzhou, China). After confirming *RET* pathogenic variants in 73 MEN2 patients or rare variants in 118 cases of sporadic MTC (sMTC), and 15 cases of sporadic PHEO (sPHEO) patients were performed. Relatives of patients were also assessed for family studies. All individuals were subjected to clinical examinations, biochemical/imaging examinations, and genetics screening according to the published criteria [1, 7] and/or underwent surgical treatment. Data of 697 individuals were summarized in Fig. 1. Most of the study population was from Zhejiang Province, and a few were from Shanghai, Guangdong, Jiangsu, Anhui, Fujian, Guangxi, Sichuan, Hubei, Henan, Shandong, Shanxi, Xinjiang, and other provinces and cities in China. The study protocol was approved by the Ethics Committee of the 903rd PLA Hospital, and written informed consent was obtained from all study subjects or their legal guardians.

### RET *screening using targeted sequencing*

Targeted sequencing using an Illumina DNA-HiSeq 2000 Analyzer was performed as previously described [7, 8, 39]. Briefly, genomic DNA was extracted from EDTA-anticoagulated peripheral blood and then used for target capture, enrichment, and elution. A custom capture array (NimbleGen; Roche) was designed to capture all exons and flanking splice junctions, as well as the immediately adjacent intron sequences for 10 genes (*RET*, *NF1, MAX, TMEM127, VHL, SDHA, SDHB, SDHC, SDHD, and SDHAF2*) associated with hereditary PHEO diseases following GeneReviews (NCBI). The results were followed by Sanger sequencing with an ABI Prism 3700 automatic sequencer (Perkin-Elmer, Fremont, CA, USA).

### Molecular characterization confirmation

The entire germline *RET* coding region, including some variable splicing and flanking regions, was analyzed (Genbank RefSeq: NM_020975.4). According to American College of Medical Genetics and Genomics (ACMG) and the Association for Molecular Pathology (AMP) Guidelines (hereafter referred to as ACMG-2015), MEN2 Database, and ATA-2015 recommendations [1, 10, 40, 41], three specific terms were used to classify or define *RET* single variants: “pathogenic variant” or “mutation” refers to those variants affecting *RET* structure and function and well-defined as causing MEN2; conversely, SNPs occurring in greater than 1% of a population or self-verified not to alter cellular functional effect were considered “benign.” *RET* SNPs observed included A45A (rs1800858), A432A (rs1800860), G691S (rs1799939), L769L (rs1800861), S836S (rs1800862), and S904S (rs1800863); a “variant of unknown significance” (VUS) were those considered “likely pathogenic,” to have “uncertain significance” (UCS), and “likely benign.” Moreover, *RET* compound mutations or variants were limited to the presence of at least one pathogenic variant and at least one other simultaneous concomitant mutation (s) or VUS (defined mutation) or a combination of two or more VUS (as VUS). However, compound mutations/variants were not classified by the ATA-2015.

### In silico analyses

In silico analyses were performed using public databases and web-based software with three different bioinformatics algorithms: Sorting Intolerant From Tolerant (SIFT), Polymorphism Phenotyping-2 HDIV (PolyPhen-2 HDIV), and Mendelian Clinically Applicable Pathogenicity (M-CAP) for missense prediction of protein functional significance of the *RET* variants. Seventeen different *RET* pathogenic point mutations were also matched within exons 10, 11, and 13–16, and a G691S SNP within exon 11 as controls reported previously [7].

### Statistical analysis

All data were analyzed with SPSS version 20.0 (SPSS Inc., Chicago, IL, USA). Measurement data confirming ordinal or normal distribution was summarized and reported as the mean ± standard deviation, mean (range), or both, and comparisons of quantitative variables were made with a Student’s *t*-test. The frequency of occurrence, percentages, and comparisons of enumeration variables were assessed by the χ^2^ or Fisher’s exact test. Age-dependent penetrance of MEN2-related MTC/PHEO was assessed with Kaplan-Meier curves, and a log-rank test was used for comparisons between curves. The test level was set *α* = 0.05, and a *P* < 0.05 was considered statistically significant.

## Results

### RET *screening and clinical information*

A total of 697 individuals were subjected to *RET* screening by targeted sequencing. Of them, 73 were index cases, and their 399 relatives were used for family studies (i.e., 245 patients with *RET* mutations and 227 of their relatives without *RET* mutations); others included 118 sMTC, 15 sPHEO, and their 92 relatives with or without rare variants (Fig. 1). The frequency of mutations and rare variants in *RET* was 72.1% (276/383) in all patients, and 16.6% (52/314) in their relatives. Of the 276 patients, 213 (77.2%) had single missense point mutations and 1 (0.4%) had a del/ins mutation (Table 1), 31 (11.2%) had rare variants (Table 2), and 31 (11.2%) had compound mutations (Table 3). In other words, 88.8% of the 276 carried pathogenic *RET* mutations, of which 87.3% had single mutations, and 12.7% had compound mutations. Among the remaining 107 patients (27.9%), 93 patients were strictly defined as sMTC, and 14 patients were classified as non-MEN2-related PHEO, all of which had no *RET* mutations/variants (Fig. 1).

**Table 1:** Distribution of *RET* single mutations in MEN2 with ATA-classified, demographic characteristics and the presence of MTC, PHEO, PHPT, CLA and HD

ATA-2015 Risk category	*RET* mutation	Nucleotide change	Family (*No, %*)	*No. a*vailable / Carriers	Sex (M/F)	MTC		PHEO		HPTH		CLA		HD	
						*No.* patient (%)	MAD (range, yrs)	*No.* patient (%)	MAD (range, yrs)	*No.* patient (%)	MAD (range, yrs)	*No.* patient (%)	MAD (range,yrs)	*No.* patient (%)	MAD (range, yrs)
ATA-MOD	C609R	c.1825 T > C	1 (1.5)	1/1	0/1	1 (100)	52	–	–	–	–	–	–	–	–
	C611Y	c.1832G > A	1 (1.5)	17/17	8/9	15 (88.2)	45.9 (23–74)	2 (12.5)	53 (37,69)	–	–	1 (6.3)	40	–	–
	C611F	c.1832-1833delGCinsTT	1 (1.5)	1/1	1/0	0 (0)	–	1	32	1	32	–	–	–	–
	C618G	c.1852 T > G	2 (3.1)	4/4	2/2	4 (100)	57.8 (37–83)	–	–	–	–	–	–	–	–
	C618R	c.1852 T > C	4 (6.2)	25/25	6/19	24 (96)	40.3 (21–60)	2 (8)	57.5 (46,69)	1	60	–	–	1(4)	6+/−
	C618S	c.1852 T > A	1 (1.5)	12/12	3/9	11 (91.2)	41.1 (24–59)	–	–	–	–	–	–	–	–
	C618Y	c.1853G > A	1 (1.5)	9/9	3/6	8 (88.9)	55.3 (35–78)	–	–	–	–	–	–	–	–
	C620R	c.1858 T > C	1 (1.5)	1/1	1/0	1 (100)	27	–	–	–	–	–	–	–	–
	C620S	c.1859G > C	1 (1.5)	1/1	1/0	1 (100)	39 (39)	–	–	–	–	–	–	–	–
	L790F	c.2370G > T	8 (12.3)	15/30^a^	11/19	14 (93.3)	46.1 (27–70)	–	–	–	–	–	–	–	–
	V804M	c.2410G > A	4 (6.2)	4/4	4/0	4 (100)	50.7 (40–69)	–	–	–	–	–	–	–	–
	S891A	c.2671 T > G	2 (3.1)	6/6	4/2	3 (50)	42 (25–62)	–	–	–	–	–	–	–	–
ATA-H	C634F	c.1901G > T	2 (3.1)	3/3	1/2	3 (100)	31.7 (8–58)	1 (33.3)	26 (26)	–	–	–	–	–	–
	C634G	c.1900 T > G	2 (3.1)	7/7	3/4	7 (100)	52.2 (43–66)	3 (42.9)	54.6 (45–66)	–	–	3 (42.9)	54 (53,54,55)	–	–
	C634R	c.1900 T > C	5 (7.7)	15/15	6/9	15 (100)	32.6 (18–65)	6 (40)	34 (21–47)	1	40	1 (6.3)	40	–	–
	C634S	c.1900 T > A	1 (1.5)	3/3	2/1	3 (100)	61.3 (51–77)	–	–	–	–	–	–	–	–
	C634W	c.1902C > G	1 (1.5)	1/1	0/1	1 (100)	34	1 (100)	34	1 (100)	34	1 (100)	34	–	–
	C634Y	c.1901G > A	22 (33.8)	69/69	37/32	69 (100)	34.3 (5.5–80)	32 (46.3)	42 (18–75)	7 (10)	30 (15–55)	–	–	–	–
ATA-HST	M918T	c.2753 T > C	5 (7.7)	5/5	2/3	5 (100)	25.4(8–34)	2 (40)	36 (22–50)	–	–	–	–	–	–
Total	19		65 (100)	199/214	95/119	189 (95.0)^b^	40.5(5.5–83)^c^	50 (25.1)	41.9 (18–75)	11 (5.5)	34.2 (15–60)	6 (5.0)	42 (34–55)^d^	1 (0.5)	NA

*MEN2* multiple endocrine neoplasia type 2, *ATA* American Thyroid Association. Risk of aggressive MTC: *MOD* moderate, *H* high, *HST* highest, *M* Male, *F* Female, *MTC* medullary thyroid carcinoma, *PHEO* pheochromocytoma, *HPTH* hyperparathyroidism, *CLA* cutaneous lichen amyloidosis, *HD* Hirschsprung’s disease, *AD* average age of diagnosis, *yrs*. years, *−* negative, *NA* not available

^a^ **T**he 15 other carriers rejected further clinical and biochemical/imaging examinations

^b^ 10 of the 199 MEN2 patients had no abnormality

^c^ The mean age at the time of MTC diagnosis in184 MEN2A patients was 38.09 ± 17.18 years

^d^ Mean age at onset of CLA was 20 yrs. (range, 12–26)

**Table 2:** Rare *RET* variants: clinical feature, population database report, in silico predictive algorithms and proposition of classification following ACMG

Exon	Nucleotide change	*RET* variant	*No*. patient /all carierrs	Age atdiagnosis	Clinical phenotype	dbSNP (NO.)	1000 Genomes (frequenc y)	ExAC (frequency)	gnomAD exomes (frequency)	SIFT	PolyPhen-2 HDIV	M-CAP	Oncogenic potential in vitro (Ref)	Ref	Classification following ACMG-2015
Single base ubstitution (missense variants)															
3	c.341G > A	R114H	2/4	51.5 (50,53)	MTC	rs76397662	0.00139776	0.00088200	0.00076690	T	B	a			Likely benign
3	c.487C > T	R163W	0/1	–	–	a	a	a	0.00000812	D	P	D			UCS
5	c.832A > G	T278A	0/1	–	–	rs541929171	0.000199681	0.00001700	0.00002487	T	B	D		55	UCS
5	c.833C > A	T278N	2/9	53.5 (48,59)	MTC	rs35118262	0.00399361	0.00209900	0.00211343	D	B	a			UCS
6	c.1226 C > A	S409Y ^b^	6/15	57.6 (41 ~ 75)	MTC ^b^	a	a	a	a	D	P	D	Potential (7)	7	Pathogenic
7	c. 1441C > G	L481V	1/1	39	MTC	rs767210575	a	0.00003300	0.00004875	D	B	D			UCS
7	c.1465G > A	D489N	2/13	51 (39,63)	MTC	rs9282834	0.00379393	0.00207300	0.00215943	T	B	a		55	Benign
8	c.1573C > T	R525W	0/3	–	–	rs545625150	0.000399361	0.00002200	0.00000961	D	P	D	Low or No (39)	32, 39	UCS
10	c.1799G > A	R600Q	1/2	41 (41)	MTC	rs377767393	a	0.00003300	0.00002079	T	B	D		52	UCS
10	c.1810G > T	A604S	2/2	45 (44,46)	MTC	a	a	a	a	T	P	D			UCS
13	c.2363 T > G	I788S	1/1	43	MTC	a	a	a	a	D	D	D			UCS
14	c.2465 T > A	V822E	0/1	–	–	a	a	a	a	D	P	D			UCS
16	c.2752A > G	M918V	1/1	69	MTC	rs377767442	a	a	a	D	P	D	Low or No (50)	15, 51,52	Pathogenic
19	c.3052C > T	L1018F	1/1	46	MTC	rs766330880	a	0.00007400	0.00005686	D	P	D			UCS
Single base substitution (synonymous variants)															
8	c.1596C > T	G532G	0/1	–	–	a	a	a	a						Likely benign
11	c.2037C > T	P679P	2/7	47.3 (26,72)	MTC	rs55862116	0.00259585	0.00113000	0.00108342						Benign
14	c.2523G > A	P841P	1/3	39	MTC	rs56195026	0.00059904	0.00012600	0.00009773						Likely benign
18	c.2844G > A	G948G	0/1	–	–	rs749196396	a	0.00000820	0.00000406						Likely benign
Double base substitution or compound variants															
19	c.3202_3203delGC insTT	A1068L	1/2	47	MTC										^d^UCS
5,2,18	c.[874G > A(;) c.200G > A(;) c.2944C > T]	V292M/R67H/ R982C (*cis*)	5/11	42 (26–70) ^c^	MTC/CCH/PHEO ^c^									12, 24	^c^pathogenic
5,7	c.[833C > A(;) c.1465G > A]	T278N/D489N (*trans*)	1/1	48	MTC										^d^UCS
11,18	c.[2945G > A(;) c.2037C > T]	R982H/P679P (*NA*)	1/1	65	MTC										^d^UCS
1,19	c.[56_58delTGC(;) c.3202_3203delGC insTT]	L19delC/ A1068L (*NA*)	1/1	36	MTC										^d^UCS
Total		23 (17)	31 /83	48.0(26–75)											

*–* negative, *dbSNP* Database of Single Nucleotide Polymorphism, *1000 Genomes* 1000 Genomes Project database, *ExAC* Exome Aggregation Consortium, *gnomAD* genome Aggregation Database, *ACMG* American College of Medical Genetics and Genomics, *Ref* References, *D* damaging, deleterious or disease_causing, *P* possibly damaging, *T* tolerated, *B* benign or polymorphism, *MTC* medullary thyroid carcinoma, *PHEO* pheochromocytoma, *CCH* C cell hyperplasia, *Ref* references, *UCS* uncertain significance

^a^ Not reported

^b^ 15 carriers with *RET*-S409Y including 1 with S409Y/P679P, *trans*, by Qi et al. reported in Ref. 7, among 6 presented with isolated MTC and/or with neck lymph node and distant metastases; 3 had elevated stimulated serum calcitonin (sCtn) or concurrent marginally elevated Ctn levels, the other remaining 6 exhibited typical Ctn/sCtn levels, and suggested as an ATA-MOD

^c^ 11 carriers with V292M/R67H/R982C from 4 families were found. Of these, 4 presented with isolated MTC/CCH (mean age 51 years; range 43–70 years), 1 presented with PHEO alone (age 26 years), the remaining 4 carriers (range 19–48 years) had no abnormality and undetectable Ctn. V292M/R67H/R982C should be considered as a weaker pathogenicity. The clinical results implied that the V292M/R67H/R982C should be considered pathogenic mutation

^d^ Clinical data showed these variants of uncertain significance

**Table 3:** Clinical presentations of patients with MEN2A and *RET* compound mutations

*RET* mutation and pattern	Nucleotide change	Family (*No.*)	Sex (M/F)	*No.* patient / All carriers	MTC		PHEO		CLA	
					*No.* patient (%)	MAD (range, yrs)	*No.* patient (%)	MAD (range, yrs)	*No.* patient (%)	MAD (range, yrs)
C634Y/V292M/R67H/R982C^a^, *trans*	c.[1901G > A(;)c.874G > A(;)c.200G > A(;)c.2944C > T]	2	0/2	2/2	2 (100)	22.5 (13,32)	–	–	–	–
C634Y/R114H, *NA*	c.[1901G > A(;)c.341G > A]	1	1/0	1/1	1 (100)	15 (15)	–	–	–	–
C634Y/D489N, *trans*	c.[1901G > A(;)c.1465G > A]	2	2/1	3/3	3 (100)	33.6 (12,34,55)	–	–	–	–
C634F/V292M/R67H/R982C^b^, *trans*	c.[1901G > T(;)c.874G > A(;)c.200G > A(;)c.2944C > T]	1	0/1	1/1	1 (100)	21 (21)	–	–	1 (100)	57
C634F/T278N, *trans*	c.[1901G > T(;)c.833C > A]	1	0/1	1/1	1 (100)	29 (29)	1 (100)	29 (31)	–	–
C634S/D489N, *NA*	c.[1900 T > A(;)c.1465G > A]	1	0/1	1/1	1 (100)	34 (34)	1 (100)	34 (34)	1 (100)	36
C634S/Q194H, *trans*	c.[1900 T > A(;)c.582G > C]	1	1/1	2/2	2 (100)	43 (39,47)	–	–	–	–
C634R/P679P*,NA*	c.[1900 T > C(;)c.2037C > T]	1	1/0	1/1	1 (100)	23 (23)	1 (100)	34 (34)	–	–
C634R/I803I, *trans*	c.[1900 T > C(;)c.2409C > T]	1	1/1	2/2	2 (100)	29.5 (27,32)	–	–	–	–
C618R/A639T, *cis*	c. [1852 T > C(;).c.1915G > A]	1	3/0	3/3	3 (100)	26 (15,25,38)	–	–	–	–
C618R/T278N, *trans*	c. [1852 T > C(;).c.833C > A]	1	1/0	1/1	1 (100)	36 (36)	–	–	–	–
C618Y/R114H, *trans*	c.[1832G > A(;)c.341G > A]	1	1/1	2/2	2 (100)	32 (24,40)	–	–	–	–
C618Y/A1105V, *trans*	c.[1832G > A(;)c.3314C > T]	1	0/2	2/2	2 (100)	53 (47,59)	–	–	–	–
C618S/R114H, *trans*	c.[1852 T > A(;)c.341G > A]	1	0/1	1/1	1 (100)	12 (12)	–	–	–	–
C620S/R114H*,NA*	c.[1859G > C(;)c.341G > A]	1	0/1	1/1	1 (100)	25 (25)	–	–	–	–
L790F/K3K, *trans*	c.[2370G > T(;)c.9G > A]	1	1/0	1/1	1 (100)	63 (63)	–	–	–	–
S891A/T278N, *trans*	c.[2671 T > G(;)c.833C > A]	1	0/1	1/1	1 (100)	44 (44)	–	–	–	–
S891A/R525W^c^, *trans*	c.[2671 T > G(;)c.1573C > T]	1	1/1	2/2	2 (100)	62 (58,66)	–	–	–	–
S891A/A1068L, *trans*	c.[2671 T > G(;)c.3202_3203delGCinsTT]	1	0/3	3/3	3 (100)	32.7 (25,33,40)	–	–	–	–
Total	19	21^d^	13/18	31/31	31 (100)	33.5 (12 ~ 66)	3 (9.7)^e^	32.3 (29 ~ 34)	2 (6.1)	46.5 (36,57) ^f^

–, negative; NA, not available; M, Male; F, Female; MTC, medullary thyroid carcinoma; PHEO, pheochromocytoma; CLA, cutaneous lichen amyloidosis; MAD, mean age of diagnosis; yrs., years

^a^ 1 of 2 patients with C634Y/V292M/R67H/R982C was reported previously in Reference 24, and the age at diagnosis of MTC was 13 yrs.

^b^ The patients with C634F/V292M/R67H/R982C presented with MTC/PHEO/CLA reported previously in Ref. 42, and age at onset of CLA was 11 yrs.

^c^ 2 patients presented MTC with S891A/R525W and associated cutaneous amyloidosis binding *OSMR* variant p.G513D reported previously in Ref. 38

^d^ Of these 31 patients from 21 families, 19 patients belong to 15 families in Table 1, the remaining 12 patients from 8 families were additive and independent

^e^ Occurrence rate of PHEO in exon 11 compound mutations was 21.4%

^f^ 2 patients presented CLA and age of onset were 11, 22 yrs., respectively

### RET *single mutations and MEN2-related phenotype*

Of the 214 patients from 65 MEN2 families with 19 different single mutations (Table 1; Fig. 1), 111 patients (51.9%) harboring *RET* mutations were classified as ATA-MOD, 98 (45.8%) were ATA-H, and 5 (2.3%) were ATA-HST. The most frequent mutations were at cysteine codon in exon 11/10 [79%; in exon 11 (45.8%; C634F/G/R/S/W/Y) and exon 10 (33.2%; C618G/Y/R/S, C620R/S, C611Y, C611F, and C609R)], followed by exon 13 (14%; L790F), whereas mutation S891A in exon 15 (2.8%), M918T in exon 16 (2.3%), and V804M in exon 14 (1.9%) represented less than 5%. MEN2A was identified in 209 patients (97.7%) from 60 families (92.3%), and MEN2B was found in 5 patients (2.3%) from 5 families (7.7%). Of the 60 families with MEN2A, 30 (50%) had classical MEN2A, 5 (8.3%) had MEN2A with CLA, 1 (1.7%) had MEN2A with HD, and 24 (40%) had familial MTC (OMIM 155240) (Table 1).

**Fig. 2:**
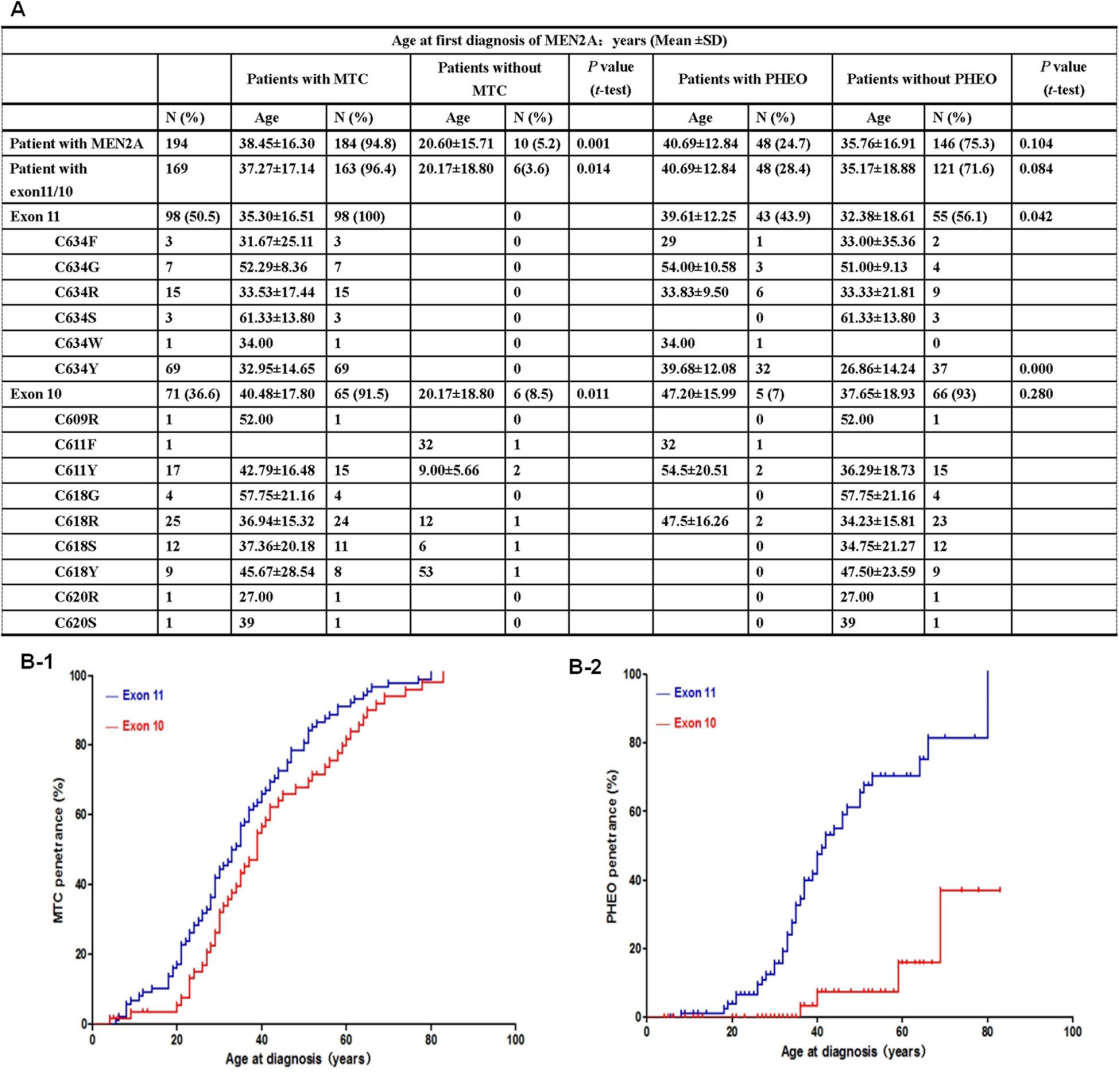
Age-dependent penetrancies of MEN2A-related MTC/PHEO carrying *RET* single pathogenic mutations. **a** Age at the diagnosis of presence or absence of MTC or PHEO for 194 MEN2A patients, for 98 patients with mutations in exon 11, and for 71 patients with mutations in exon 10, respectively. Student’s *t*-test was used to compare mean of age (mean ± SD). **b** Of 169 patients, Kaplan-Meier estimates of MTC (shown in **b-1**) or PHEO (shown in **b-2**)-specific presence in 98 patients carrying mutations in exon 11 versus 71 patients carrying mutations in exon 10. The log-rank test was used to compare curves, *P* = 0.041 or *P* = 0.000, respectively

Associations between *RET* mutation genotype and disease phenotype showed that clinical information was available at the time of molecular diagnosis for 194/199 patients with MEN2A. Of them, 68.5% had MTC alone, 17.5% had MTC and PHEO, 5.2% had MTC/PHEO and HPTH, 3.6% had MTC/PHEO and CLA or HD, 0.5% had PHEO and HPTH, and 4.7% had no abnormality. Moreover, 94.8% of the 194 patients with MEN2A presented with MTC (i.e., 100% of 98 patients with an ATA-H mutation [C634] and 89.6% of 96 patients with an ATA-MOD mutation had MTC). The mean age at the time of MEN2A diagnosis was significantly different between patients with or without MTC (38.45 ± 16.30 versus 19.33 ± 15.71 years; *t* = 3.43, *P =* 0.001; Table 1, Fig. 2a). Also, 25.1% of the 194 patients presenting with at least 1 PHEO had all mutations occur in exons 11/10 and only involved cysteine residues. The mean age at the time of MEN2A diagnosis did not significantly differ between patients with or without PHEO (40.69 ± 12.84 versus 35.76 ± 16.91 years; *t* = 1.391, *P* = 0.104; Table 1; Fig. 2a).

Also, 96.4% of the 169 patients with mutations in exons 11/10 presented with MTC, and only 6 patients with exon 10 mutations had no MTC. The mean age at the time of MEN2A diagnosis was significantly different between patients with or without MTC (37.27 ± 17.14 versus 17.80 ± 19.99 years; *t* = 2.420, *P =* 0.014). Comparison of Kaplan–Meier curves revealed the proportion of MEN2A patients with MTC was significantly different between patients with exon 11 and 10 mutations (*P* = 0.041, log-rank; Fig. 2a, b-1). Further analysis of the 48 patients with PHEO (28.4%) of the 169 patients revealed that 43 carried mutations in exon 11 (43.9% out of 98 patients, C634F/G/R/S/W/Y), while 5 carried mutations in exon 10 (7.0% out of 71 patients, C611Y/C611F/C618R) [*P* = 0.000 for exon 11 versus 10; Table 1; Fig. 2a]. Moreover, comparison of Kaplan–Meier curves revealed PHEO penetrance in MEN2A was significantly different between patients with mutations in exons 11 and 10 (*P* = 0.000, log-rank; Fig. 2a, b-2). The most frequent mutation was C634Y (66.6%), followed by C634R (12.5%), C634G (6.2%), C618R (4.2%), C611Y (4.2%), C634F (2.1%), C634W (2.1%) and C611F (2.1%), respectively (Table 1; Fig. 2a). The mean age at diagnosis was significantly different between patients carrying a C634 mutation with or without PHEO (39.61 ± 12.25 versus 32.38 ± 18.61 years; *t* = 2.068, *P* = 0.042). In exon 10, the mean age at diagnosis was 47.20 ± 15.99 versus 37.65 ± 18.93 years and did not significantly differ (*t* = 1.090, *P* = 0.280; Fig. 2a). Also, the mean age of PHEO diagnosis did not significantly differ between patients with mutations in exon 11 or 10 (39.61 ± 12.25 versus 47.20 ± 15.99 years; *t* = 1.261, *P* = 0.215; Fig. 2a). However, the mean age of PHEO diagnosis for those with a mutation in exon 10 was relatively later (by 7.6 years). Moreover, bilateral PHEO occurred synchronously or metachronously in 52.1% of 48 patients; 25 carried an exon 11 mutation (25/43), and 0 carried a mutation in exon 10 (0/5) [*P* = 0.014]. MTC was diagnosed before PHEO in 17 patients (35.4%), synchronously in 21 (43.8%), after PHEO in 9 (18.7%), and 1 (2.1%) with synchronously PHEO/HPHT. In addition, HPTH and CLA had relatively low prevalence and were mainly described in patients with a C634 mutation (HPTH, 9 cases; CLA, 5 cases); otherwise, 2 case had HPTH in C618R and C611F mutations, and 1 had a C611Y mutation with CLA. HD was only present in 1 patient with a C618R mutation. In the above 11 HPTH cases, 10 were diagnosed concurrently with MTC. Conversely, in the 6 cases with CLA, the mean age at onset of CLA was 20 years (range, 12–26 years), younger than the average age at diagnosis of CLA (42 years; range, 34–55 years), MTC (40.53 years; range, 5.5–83 years), and PHEO (42.1 years; range, 18–75 years) [Table 1]. On the other hand, of the 5 cases of MEN2B with an M918T mutation (de novo mutation in 3 patients), 2 had MTC, 2 had MTC and PHEO, 1 had mixed medullary-follicular carcinoma, and all presented with a typical facies, numerous mucosal neuromas in the anterior tongue, lips, and buccal mucosa, and a Marfanoid habitus (Table 1).

### *Rare* RET *variants and clinical features*

The presence of 23 rare variants, 18 single variants (14 missense and 4 synonymous), 1 double base substitution, and 4 compound variants not classified by the ATA-2015 was found in 83 carriers in this series (Table 2). Seventeen of these were identified in 75 carriers present in 31 patients, and the mean age at MTC diagnosis was 49 years (range, 26–75 years). Of them, 30 patients only with MTC, 1 patient had PHEO alone. Among 25 patients were diagnosed with MTC after age 40, whereas 6 were diagnosed with MTC or PHEO before age 40 (Table 2; Fig. 1).

Of these 18 single variants, 10 missense variants associated with MTC, 3 (I788S, S409Y, and A604S) had not been previously described in the Database of SNP (dbSNP), 1000 Genomes Project (1000 GP), Exome Aggregation Consortium (ExAC), or genome Aggregation Database exomes (gnomAD exomes) (Table 2)*.* The I788S (c.2363 T > G), was considered a “damaging” variant by three different in silico analyses, and a patient presenting with MTC alone was diagnosed at 43 years-old. Variant S409Y (c.1226C > A) also qualified as “damaging” or “possibly damaging,” and 5/14 carriers were diagnosed with MTC at a mean age of 57.6 years (range, 41–75 years). The latter, A604S (c.1810G > T), qualified as “tolerated,” “possibly damaging,” or “damaging” depending on the 3 algorithms used; 2 patients from 2 different families were diagnosed with MTC at 44 and 46 years-old, respectively. Of the remaining 7 missense variants associated with MTC listed in these databases (Table 2), 2 variants [M918V (c.2752A > G) and L1018F (c.3052C > T)] qualified as “damaging” or “possibly damaging,” and 2 patients were diagnosed with MTC at 69 and 46 years-old, respectively. The other 5 variants qualified as being either “damaging” or “benign;” 2 of these 8 patients with MTC were diagnosed before age 40. Three variants, R114H (rs76397662), T278N (rs35118262), and D489N (rs9282834), and the following compound mutations described, appeared at a relatively high frequency (Tables 2, and 3). Also, the frequencies in East Asian populations listed in the 1000 GP, ExAC, and gnomAD exomes databases were relatively higher than that of other ethnic populations (https://www.pubvar.com/variant/10-43597793-G-A; 43,600,607-C-A; 43,606,856-G-A). Interestingly, two synonymous variants, P679P (c.2037C > T) in 2 patients with MTC were diagnosed at 26 and 72 years, and P841P (c.2523G > A) in 1 patients with MTC was diagnosed at 39 years-old, respectively, were found in 10 carriers from 4 families. The remaining 6 single variants, 4 missense and 2 synonymous, were found in 8 carriers who had no evidence of MEN2-related clinical manifestation (Table 2). Further, InterVar Classify System which mainly consists of automatically interpretation by 28 criteria and manual adjustment by users to re-interpret the clinical significance was used for classifying all these 18 single variants according to the consensus recommendation of the ACMG-2015 (http://wintervar.wglab.org/). Two variants (S409Y and M918V) could be classified as pathogenic, 10 of UCS, 4 of likely benign, and 2 as benign (Table 2).

Moreover, a double base substitution variant, A1068L (c.3202_3203delGCinsTT), was found in a female diagnosed with MTC at 47 years. Nine patients had 4 different compound variants, of which 5 patients from 4 families carried *cis* V292M/R67H/R982C (3 had MTC at 43, 47, and 70 years, respectively; 1 had C cell hyperplasia in thyroid at 44 years; and 1 had left PHEO alone at 26 years). One patient carrying *trans* T278N/D489N was diagnosed with MTC at the age of 48. The remaining isolated 2 patients carrying R982H/P679P or the new compound variant L19delC/A1068L had MTC at 65 and 36 years-old, respectively. These clinical data implied the results that V292M/R67H/R982C was considered as a pathogenic mutant, and the other 3 of UCS.

### RET *compound mutations and MEN2A-related phenotypes*

Nineteen compound mutations in 31 patients from 21 families were found, including 19 patients belonging to 15 families in Table 1 and 12 patients from 8 families that were additive and independent. The mean age at MTC diagnosis was 34.58 ± 15.82 years, whereas the MTC diagnostic age of 184 patients with single mutations and MEN2A was 38.09 ± 17.18 years (Table 1). While these ages did not significantly differ (*t* = 0.826, *P* = 0.410), there seems to be a trend towards younger onset (Table 3).

Of the 14 patients carrying exon 11 compound mutations, 2 with concomitant *trans* C634Y/V292M/R67H/R982C mutations were diagnosed with MTC at 13 and 32 years, respectively, and 1 harboring a *trans* C634F/V292M/R67H/R982C mutation presented with MTC at 21 years-old, diagnosed with CLA at age 57. The other 7 patients with *trans* C634Y/D489N, C634S/Q194H, or C634R/I803I mutations were diagnosed with MTC at a mean age of 35.1 years (range, 12–55 years). Moreover, 1 patient carrying a C634Y/R114H mutation presented with MTC alone at age 15; 1 carrying C634F/T278N and 1 with C634R/P679P were diagnosed with MTC at 29 and 23 years-old, respectively, as well as PHEO at 29 and 34 years-old; and 1 carrying C634S/D489N was diagnosed with MTC/PHEO at age 34 and CLA at age 36. Unfortunately, no family study was available for these 4 patients. The MTC and PHEO diagnostic ages of 14 patients carrying exon 11 compound mutations was 29.50 ± 12.43 and 35.30 ± 16.51 years, respectively, while those of 98 patients carrying single mutations were 33.25 ± 1.50 and 39.61 ± 12.25 years, respectively. The occurrence of PHEO in patients with exon 11 compound mutations and single mutations was 28.6% (4/14) and 39.8% (39/98), respectively, and there were no significant differences between the three (*t* = 1.258, *P* = 0.211; *t* = 1.026, *P =* 0.311; *P =* 0.429; Table 3; Fig. 2a).

The other 10 patients with exon 10 compound mutations all had MTC alone; 6 carried *trans* C618R/T278N, C618Y/R114H, or C618Y/A1105V mutations and were diagnosed at a mean age of 36.3 years (range, 12–59 years). Another male patient with C618R/A639T mutation was diagnosed with MTC at age 38. His son and daughter, carrying a C618R/A639T mutation, respectively, were respectively diagnosed with MTC at 15 and 25 years-old, however, his wife had non-C618R or A639T, meaning the C618R/A639T mutation was *cis*. In addition, an isolated female patient with a 620S/R114H mutation was diagnosed at age 25. The MTC diagnostic ages of 10 patients with exon 10 compound mutations (32.1 ± 14.61 years) and 65 patients with single mutations (40.48 ± 17.80 years) were not significantly different (*t* = 1.401, *P* = 0.166). The remaining 7 patients presenting with MTC alone and *trans* L790F or S891A compound mutations were diagnosed at a mean age of 41.8 years (range, 16–66 years). This included 3 patients carrying an S891A/A1068 mutation diagnosed at 25, 33, and 40 years, respectively.

### *Additional information and* RET *polymorphisms*

**Fig. 3:**
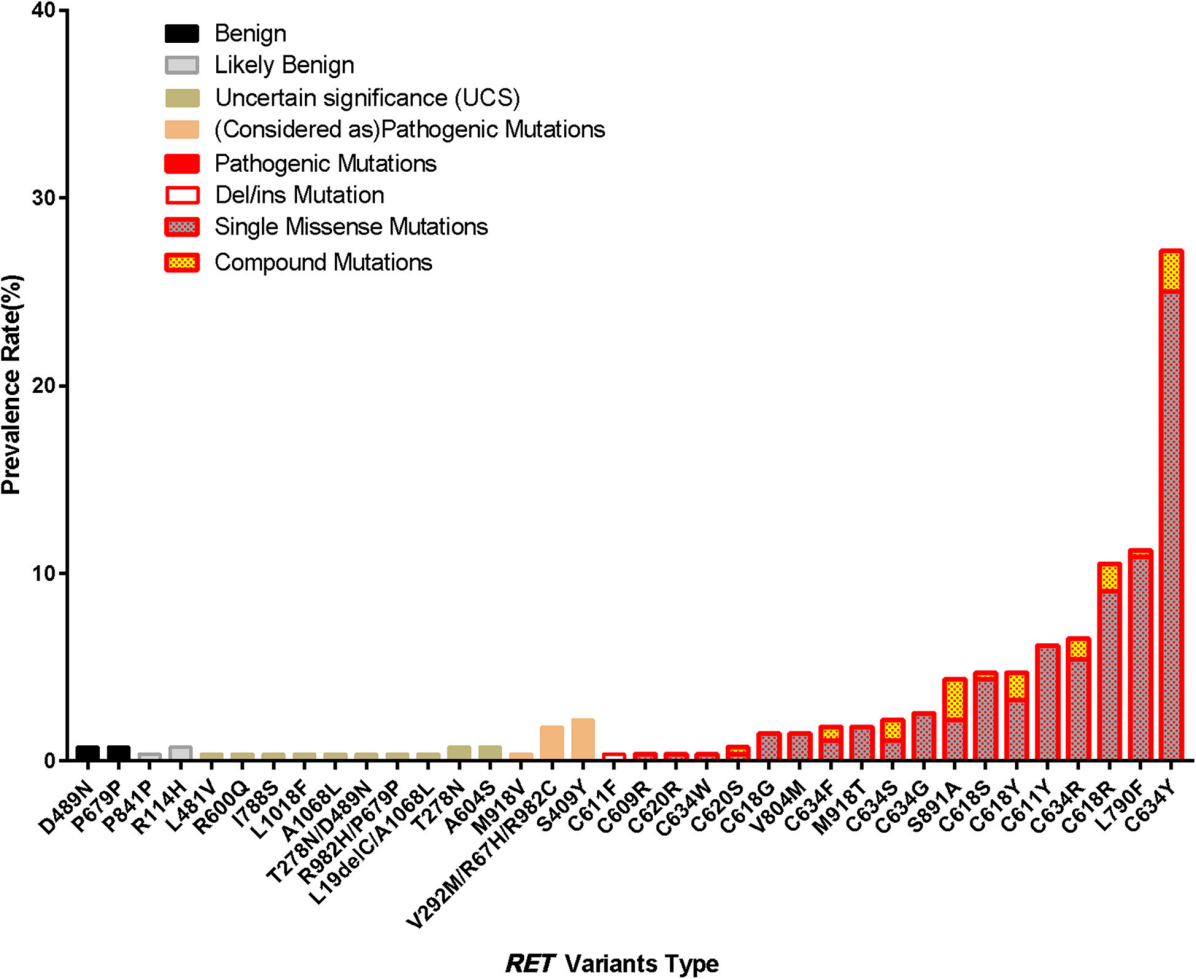
Prevalence along the germline *RET* mutations/variants in our series. Fifty-five different variants of *RET* were found in 276 patients with confirmed or suspected MEN2. Of them, 38 were pathogenic including 19 (18 point and 1 del/ins) single and 19 compound mutations in 245 patients. The remaining 17 variants in 31 patients, 3 were considered as pathogenic variants in 12 patients, other 12 variants of unknown significance (VUS; 10 of uncertain significance and 2 of likely benign) or 2 as benign in 19 patients

A total of 328 carriers with 61 different *RET* variants including 38 pathogenic and 23 variants were found (Tables 1, 2 and 3; Fig. 1). With the exception of 6 *RET* variants in 8 carriers that exhibited no evidence of MTC/PHEO, 55 different *RET* variants (38 pathogenic and 17 variants) in 276 patients with confirmed or suspected MEN2 are shown in Fig. 1 and Fig. 3, and distribution of pathogenic variants frequencies is summarized in Table 4. Interestingly, 5 offspring of individuals with MEN2A-related unilateral PHEO were diagnosed at 18, 21, 26, 29, and 37 years. So far, their father or mother with MEN2A showed no clinical, biochemical, or imaging manifestations of PHEO. The frequency in all 51 patients with MEN2A-related PHEO was approximately 10% (Tables 1, 3, and 5). Moreover, family studies unexpectedly found that 5 index patients (probands) belonging to 5 families were adopted as an orphan or abandoned child (3 C634Y, 1 C634S, and 1 C634W mutations), while their foster parents and/or siblings without *RET* mutations consistently showed no evidence of MEN2A. As a consequence, 6.8% of the 73 *RET*-defined families were actually adoptive families (Tables 1 and 3) in addition to 1 patient with an S409Y mutation that was not classified by the ATA-2015 which was also adopted. The presence of 5 *RET* SNPs (A45A, A432A, G691S, L769L, and S904S) and absence of S836S (rs1800862) was observed in all 697 individuals included, similar to those shown in East Asian populations listed in the 1000 GP, ExAC, and gnomAD exomes databases. The S836S mutation frequency was lower than that of other ethnic populations (https://www.pubvar.com/variant/10-43615094-C-T). Whether the SNP alleles or haplotypes observed in the present series are involved in MEN2 pathology or play increase susceptibility for MTC/PHEO needs to be further described elsewhere.

**Table 4:** Distribution of germline RET pathogenic mutation frequencies observed in China and in 5 major published studies, BrasMEN (17), France (16), Germany (15), ItaMEN (18) and Greece (43). The table references to Lebeault et al. (16) and Rui et al. (17) reported previously and complements our data

*RET* codon	*NO.* of families (%)					
	China*	BrasMEN	France	Germany	ItaMEN	Greece
V292M^§^	4 (4.9)					
T388					1 (0.4)	
S409Y	4 (4.9)					
C515			2 (0.5)		1 (0.4)	
dup531			1 (0.2)			
G533		1 (0.6)	3 (0.7)			21 (36.2)
K603			1 (0.2)			
C609	1 (1.2)	7 (4.0)	5 (1.1)	1 (0.5)	6 (2.4)	
C611	2 (2.4)	6 (3.4)	12 (2.7)	6 (3.1)	1 (0.4)	
C618	10 (12.2)	6 (3.4)	29 (6.5)	11 (5.8)	15 (6.1)	4 (6.9)
C620	3 (3.7)	6 (3.4)	31 (7.0)	14 (7.3)	9 (3.7)	5 (8.6)
C630		1 (0.6)	1 (0.2)	1 (0.5)	4 (1.6)	
C634	35 (42.7)	76 (43.2)	44 (32.4)	73 (38.2)	86 (35.1)	19 (32.8)
S649			2 (0.5)			
K666			7 (1.6)		1 (0.4)	
E768		6 (3.4)	14 (3.2)	2 (1.0)	9 (3.7)	1 (1.7)
L790	10 (12.3)	3 (1.7)	43 (9.7)	26 (13.6)	8 (3.3)	
V804	4 (4.9)	23 (12.5)	95 (21.4)	19 (9.9)	52 (21.2)	3 (5.2)
M848					1 (0.4)	
A883			3 (0.7)		1 (0.4)	
S891	3 (3.7)	8 (4.5)	20 (4.5)	6 (3.1)	23 (9.4)	
S904					1 (0.4)	
R912			1 (0.2)			
M918T	5 (6.1)	26 (14.8)	29 (6.5)	32 (16.8)	20 (8.2)	5 (8.6)
M918V	1 (1.2)	8 (4.5)	1 (0.2)			
No mutations					6 (2.4)	
**Total**	**82 (100)**	**168 (100)**	**444 (100)**	**191 (100)**	**245 (100)**	**58 (100)**

^§^V292M, Refer in particular to the V292M/R67H/R982C

*Our these 82 families included 72 families with single missense mutation or compound mutations, 1 family with del/ins mutation and other 9 families respectively with V292M/R67H/R982C, S409Y or M918V considered as pathogenic mutations

**Table 5:** Five offspring of individuals with MEN2A-PHEO and their parents with MEN2A no evidence of PHEO and *RET* variants/SNPs

Family	*RET* mutation	Age of diagnosis (years)*	Phenotype	PHEO, L/R (cm)	Concomitant of *RET* variants/SNPs
1	C634Y	Son, 18	MTC + PHEO	−/5.5	A45A, A432A, L769L
		Father, 42	MTC	–	A45A, A432A
2	C634R	Son, 21	MTC + PHEO	3.5/ –	A45A, A432A, G691S, L769L
		Father, 47	MTC	–	A432A
3	C634Y	Daughter, 26	MTC + PHEO	−/4.0	A432A
		Mother, 55	MTC	–	A45A, A432A
4	C634F	Daughter, 29	MTC + PHEO	2.7/ –	A45A, T278N, A432A
		Mother, 57	MTC + CLA	–	V292M, R67H, R982C, A432A, L769L, S904S
5	C611Y	Daughter, 37	MTC + PHEO+CLA	−/4.8	A45A, A432A, L769L
		Father, 74	MTC	–	A45A, A432A, L769L

*The age at which the offspring was diagnosed with unilateral PHEO and the age of their father or mother of MEN2A patient at that time

MTC, medullary thyroid carcinoma; PHEO, pheochromocytoma; CLA, cutaneous lichen amyloidosis; **L/R, left or right;** SNPs, **single nucleotide polymorphisms**

## Discussion

To our knowledge, the present study is the first to analyze the distribution of *RET* mutations/variants in confirmed or suspected MEN2 patients by targeted sequencing in an ethnic Han Chinese cohort (Table 1; Fig. 1). With the exception of 93 patients defined as sMTC and 14 classified as non-MEN2-related PHEO, 55 different pathogenic or variants of *RET* were found in 276 patients with confirmed or suspected MEN2 (Tables 1, 2 and 3; Fig. 1). C634 in exon 11 was the most frequently mutated codon. However, there are differences in the frequency of these mutations due to sample size, geography, and ethnicity. For example, C634 has been found in approximately 30.6–43.2% of European-Americans [14–17] and 37.1–72.5% of Asian populations [11, 43]. Moreover, the G533C mutation, with a prevalence of 36.2% in Greece, has also appeared to cluster in Brazilians living in the US and Mediterranean countries that might be extrapolated to originate from Greece, but rarely reported in other ethnic groups [Table 4] [13–17, 43–45]. In the current series, the high prevalence of families with single mutations that were carriers of C634Y (33.8%) was presumably caused by a founder effect*.* Conversely, the frequency of families with V804M (4.9% ~ 6.2%) was lower than that reported in France and Italy but similar to that in Greece [Tables 1 and 4] [14, 16, 18]. These results implied that the spectrum of *RET* mutations identified in the present study is quite different from that found in other countries [Fig. 3; Table 4] [14–17, 43–45].

Over the past 25 years, new insight into the natural course of disease and genotype-phenotype data caused a paradigm shift in management of MEN2. Identification of asymptomatic carriers of *RET* mutations in exons 8, 10, 11, and 13–16 by a routine procedure, followed by appropriate screening through pedigree investigation, early diagnosis, and timely prophylactic treatment is essential to improving the likelihood of good outcomes [1, 9, 21–24]. In present study, 88.8% of 276 patients presenting with MEN2 carried *RET*-defined mutations, of which 87.3% had single mutations, and 12.7% had compound mutations. However, 31 (11.2%) patients with suspected MEN2 carried *RET* variants mostly located in non-hotspots and were not classified by the ATA-2015 (Fig. 1; Tables 1, 2 and 3), which is consistent with recent reports of more non-cysteine-linked codon mutations and rare *RET* variants [14, 20]. Nonetheless, these results provide a novel insight into MEN2 development.

Nineteen single mutations of *RET* in 214 patients with MEN2 were found herein (Table 1; Figs. 1, 2, and 3). Of these, 18 were involved in the development of MEN2A- related MTC in 94.8% of 194 carriers with available clinical information. Of which 98 patients with C634 mutations (ATA-H) all presented with MTC. In accordance with the ATA-2015 recommendation, prophylactic thyroidectomy should be performed before age 5, whereas 10/96 patients with an ATA-MOD mutation and normal basal serum calcitonin levels should be actively followed up and monitored [*P* = 0.001] [1, 4, 7, 18]. Also, 87.1% of the 194 patients carrying mutations in exons 11/10, and 24.7% of patients presenting with PHEO only associated with cysteine mutations in these two exons, predominantly in C634 mutations with a combined PHEO was 43.9%, and the youngest PHEO diagnostic age was 18. The evidence seems to suggest that clinical follow-up of MEN2A cases with non-cysteine mutations could be simplified by eliminating the annual or extending times interval evaluation of PHEO, which is still recommended by guidelines. Of course, it supports that mandatory in patients with cysteine mutations, in particular at C634 [1, 14–18, 36]. Further analysis of the estimated cumulative frequency of MTC or PHEO by current Kaplan-Meier curves showed that penetration rates of MTC and PHEO were higher in patients with mutations in exon 11 versus 10 (*P* = 0.041 and *P* = 0.000, respectively), and PHEO incidence rates in patients with mutations in exon 11 were higher than in those with mutations in exon 10 (*P* = 0.000). Moreover, these bilateral PHEO were always associated with exon 11 mutations (*P* = 0.014) and lack of non-cysteine *RET* mutations (Table 1; Fig. 2). In contrast, the mean age at PHEO diagnosis was not significantly different between patients with exon 11 and 10 mutations (*P* = 0.215), though those with exon 10 mutations occurred relatively later 7.6 years. Of note, 5 offspring of individuals with MEN2A-related PHEO, but their father or mother with MEN2A had consistently no evidence of PHEO. These unexpected findings indicate that patients presenting with the same genetic alteration can vary significantly in clinical phenotype. It is speculated that the natural history of MEN2A-related PHEO could be influenced by genetic or environmental modifying factors, but continued research to confirm or refute this hypothesis is necessary, especially with respect to *RET* variants/SNPs seemingly not involved in the development of PHEO in these specific 5 patients [1, 16, 27, 30, 36, 38] (Table 5). Moreover, in the present study, exception with a genotype-phenotype relationship between MEN2A-related CLA and *RET* C611Y [45], the CLA and HPTH mainly involving C634, in contrast to be classically reported that CLA develops in up to 9% of carriers, HPHT in 20–30%, and HD in 7%, suggesting those prevalence rates in Chinese or Asian populations were lower than that in European-Americans [1, 11, 43]. Even so, CLA might present earlier, prior to the onset of clinical symptoms, facilitating early recognition of individuals at risk of MEN2A-specific tumors [1, 46]. The remaining 1 single mutation involved in the development of MEN2B in 5 patients, all of whom were affected by the M918T mutation that it is known to present a unique physical appearance characteristic of extra-endocrine signs. However, the frequency and prevalence rate of the de novo M918T mutation in the present study were lower than those reported previously [60% versus 90 and 2.3% versus 5%, respectively] [1, 47]. Unexpectedly, 1 patient with MEN2B presented with mixed medullary-follicular carcinoma in conjunction with special immunostaining features, which may be unique biological behavior and a relatively favorable prognosis relative to other MEN2B-related pure MTC [48].

Herein, 17 rare *RET* variants were found in 75 carriers present in 31 patients with suspected MEN2 mostly diagnosed after age 40 (Table 2; Fig. 3), and the majority of these variants have uncertain biological significance. However, it is interesting that an I788S variant found in a 43-year-old patient with MTC was predicted to be “damaging”, while the substitution is considered to be of UCS according to ACMG-2015 guidelines. In addition, a 46-year-old patient with MTC was reported to have the synonymous heterozygous variant I788I [c.2364C > T] [49], implying that I788S may be a UCS or a potentially pathogenic mutation. Two patients diagnosed at 44 and 46 years, respectively, had an A604S variant classified as UCS depending on the 3 algorithms used and following the ACMG-2015. An important finding of the present series was the discovery of the S409Y variant classified as “damaging” or “possibly damaging” whose functional tests had a low oncogenic potential. Meanwhile, cosegregation with MTC in at least 6 patients of 4 families affected by S409Y causing the disease has been definitively confirmed [7]. The S409Y variant is considered a pathogenic variant by the ACMG-2015. The M918V mutation has been shown to have low oncogenic potential by in vitro testing [50] and has been associated with a moderate risk of MTC in a recent study of multiple families, none of whom presented clinical features of MEN2B [16, 51]. Herein, the patient with M918V was diagnosed with MTC alone at age 69, further supporting that M918V may induce in MTC, and is classified as a pathogenic mutation by the ACMG-2015. Thus, S409Y and M918V variants should be classified as ATA-MOD mutations associated with familial MTC [Fig. 3; Table 4] [7, 16, 51]. In contrast, a 41-old-year female patient carrying R600Q had MTC, but her father carrying R600Q had no abnormality, similar to a previous report [52], classifying this variant as UCS. Interestingly, a relatively high frequency of R114H, T278N, and D489N variants was revealed in the current series and in other East Asian populations, mainly associated with HD, but rarely reported to play a role in MTC or in tumorigenesis [53–56]. Herein, patients with these 3 variants had no family history and alterations that were more like sMTC. Currently, limited evidence is available regarding whether or not these 3 variants are “benign”. Five patients from 4 families were found to have the *cis* V292M/R67H/R982C compound mutation. One had isolated MTC at 70 years-old, and 1 had C cell hyperplasia at 44 years-old. Of the other 3 from 3 families, 2 had MTC at ages 43 and 47, and 1 had PHEO alone at age 26. Meanwhile, another study reported an Italian MEN2A patient with V292M presented at the age of 44 with PHEO and MTC, and in vitro assays showing a low-grade transforming potential associated with V292M have been reported previously [57]. In this study, the V292M/R67H/R982C alteration was considered to have weaker pathogenicity [Fig. 3; Table 4] [13, 25, 57]. There are no family studies available on the other variants diagnosed before age 40 and rare variants (A1068L and L19delC/A1068L), so their influence on the clinical course of MTC is not yet clear. Similarly, the fact that the remaining 6 variants (4 of UCS and 2 of likely benign) were found in 8 carriers even though none of them had MEN2 remains to be further clarified (Table 2). Nonetheless, accurate characterization of the pathogenic role of these variants, including new family studies, and the correlation between genotype-phenotype would be of great relevance for patients. Clinicians should be prudent in trusting in silico results and choose appropriate treatment approaches for patients with susceptibility variants.

Regarding the discovery of the 19 compound mutations in 31 patients with MEN2 (Table 3; Figs. 1, and 3), all major mutations were classified as either ATA-H or ATA-MOD, whereas secondary variants were not classified by the ATA-2015. The mean age at MTC diagnosis of those with compound mutations showed a trend towards younger ages relative to those with single mutations, and there seems to be no need to change the risk grade. However, compound mutations C634Y/V292M/R67H/R982C and C634F/V292M/R67H/R982C seemed to lead to higher MTC aggressiveness or clinical staging than C634Y or C634F and V292M/R67H/R982C [25, 42], while the compound mutations S891A/A1068L and C618R/A639T may predispose to a relatively earlier MTC diagnostic age than that of S891A and C618R. Thus, an additive synergistic effect is speculated to exist. In contrast, 2 patients with S891A/R525W mutations showed a relatively later MTC diagnostic age than those with S891A described here and elsewhere [37, 58]. These results reflect the fact that the combined secondary variant has a duality or diversity of effects on disease genesis, although there is insufficient clinical data in the present study to support a change in risk category. Since 12 patients with concomitant secondary variants R114H, T278N, and D489N presented with a relatively wide range of diagnostic ages, the clinical disease course remains uncertain and inconsistent. A cautious approach might be to remain aware of the possible concomitant presence of compound mutations when faced with an abnormal natural disease course or unusual clinical features; however, appropriate validation is needed to avoid misinterpretation and irreversible clinical consequences [25, 29, 30, 37, 38, 42].

Approximately, 5.6–9% of patients with MEN2A are de novo *RET* mutation, and that almost always arises from the paternal allele [59]. However, there are no MEN2A patients carrying de novo mutations in the present series. Screening for germline *RET* mutations in new patients with MTC regardless of their family history is recommended and may help not only to discover de novo families, but also correctly diagnose approximately 1–7% of patients with presumed sMTC that actually have MEN2 [1, 14, 19], such as patients with S409Y, M918V, and C609R, C634W in the present series. Unexpectedly, however, at least 6.8% of families in the current study were found to be adoptive, reflecting a thought-provoking socioeconomic phenomenon—relatively high abandonment and/or adoptive rates of children with MEN2A, more likely to occur in emerging countries [1, 19, 59]. Controversially, data of adopted children was usually protected and managed by various legislative and social regulations, the worldwide adoptive rate might need further clarifying. Integrate accurate genealogical information, *RET* testing, and statement records of proband patients, which could have a cautious and clinical diagnosis of being adoptive. Considering public policy standpoints and social ethics, cost-effective haplotype analysis seems unnecessary. Nevertheless, an accurate diagnosis depends on various molecular biology methods, and haplotype analysis is still worth performing to determine lineage, facilitating a biological definition for adoptive families [1, 25, 59]. Additionally, the SNP S836S was absent in the present study, similar to observations in East Asian populations showing its frequency was lower than that of other ethnic populations.

## Conclusions

In conclusion, the present data are largely consistent with the current evidence- based recommendations in the European-American clinical practice guidelines. Since the genetic, socioeconomic, and environmental backgrounds of Asian populations is very different, diversity in *RET* variants or population carriers may result in a different natural disease course. Further large-scale studies using targeted sequencing should be conducted as it is a rapid, accurate and cost-effective approach for study of genotype-phenotype correlations and discovery of rare or unknown variants of *RET*.

## Data Availability

The dataset used and/or analyzed during the current study are available from the corresponding author on reasonable request.

## Abbreviations

MEN2: Multiple endocrine neoplasia type 2
MTC: Medullary thyroid cancer
PHEO: Pheochromocytoma
HPTH: Hyperparathyroidism
HD: Hirschsprung’s disease
CLA: Cutaneous lichen amyloidosis (CLA)
ATA: American Thyroid Association
SNP: Single nucleotide polymorphisms
ACMG: American College of Medical Genetics and Genomics
AMP: Association for Molecular Pathology
VUS: Variant of unknown significance
UCS: Uncertain significance
SIFT: Sorting Intolerant From Tolerant
PolyPhen-2 HDIV: Polymorphism Phenotyping-2 HDIV
M-CAP: Mendelian Clinically Applicable Pathogenicity
dbSNP: Database of SNP
1000 GP: 1000 Genomes Project
ExAC: Exome Aggregation Consortium
gnomAD exomes: Genome Aggregation Database exomes
